# Supplementary material for: From cell lines to cancer patients: personalized drug synergy prediction
Source: Bioinformatics. 2024 May 8;40(5):btae134. doi: 10.1093/bioinformatics/btae134 (PMC11215552; doi:10.1093/bioinformatics/btae134)
Supplement: btae134_Supplementary_Data [file btae134_supplementary_data.pdf]

Supplementary Material for From Cell-Lines to Cancer Patients: Personalized Drug Synergy Prediction

Halil Ibrahim Kuru[1], A. Ercument Cicek[1,2,*] and Oznur Tastan[3,*]

1. Department of Computer Engineering, Bilkent University, Ankara, Turkey 06800
2. Computational Biology Department, Carnegie Mellon University, Pittsburgh, PA 15213
3. Faculty of Engineering and Natural Sciences, Sabanci University, Istanbul, Turkey 34956

*Correspondence: cicek@cs.bilkent.edu.tr and otastan@sabanciuniv.edu

1 Supplementary Tables

1.1 Top Cell Line Performances for Different Models

Table 1. Top 10 cell line performance for PDSP

Cell line	# of occurrences in training data	MSE	Pearson	Spearman
OVCAR-4	2934	10.56	0.59	0.48
SNB-75	2822	11.56	0.65	0.49
NCI-H226	2991	13.37	0.60	0.48
LOVO	365	13.56	0.40	0.44
A427	365	13.70	0.68	0.66
TK-10	2962	14.10	0.67	0.50
UWB1289	365	14.23	0.56	0.54
OV90	365	14.30	0.57	0.65
EKVX	2876	14.72	0.60	0.51
OVCAR-5	2970	14.92	0.66	0.53

Table 2. Top 10 cell line performance for MatchMaker

Cell line	# of occurrences in training data	MSE	Pearson	Spearman
OVCAR-4	2934	10.66	0.59	0.46
SNB-75	2822	12.03	0.63	0.47
LOVO	365	13.25	0.38	0.43
UWB1289	365	13.51	0.56	0.56
NCI-H226	2991	13.79	0.58	0.46
TK-10	2962	14.52	0.66	0.49
OV90	365	14.75	0.54	0.60
A427	365	15.04	0.64	0.66
SK-OV-3	3301	15.68	0.62	0.52
OVCAR-5	2970	15.78	0.63	0.53

Table 3. Top 10 cell line performance for DeepSynergy

Cell line	# of occurrences in training data	MSE	Pearson	Spearman
OVCAR-4	2934	12.30	0.52	0.36
OV90	365	14.46	0.58	0.60
LOVO	365	14.67	0.28	0.31
SNB-75	2822	14.75	0.53	0.41
UWB1289	365	15.47	0.52	0.53
NCI-H226	2991	16.70	0.48	0.37
PC-3	2944	17.35	0.46	0.40
A498	3059	17.50	0.54	0.44
EKVX	2876	18.33	0.51	0.41
OVCAR-5	2970	18.51	0.59	0.49

1.2 Chemical Features

Drug chemical structure features are calculated by a Python library called *ChemoPy*. The chemical features includes 11 different chemical descriptor types with a total of 541 different features. Table 4 shows the chemical feature types and corresponding number of features.

Table 4. Drug chemical structure features collected from ChemoPy

Chemical Feature Type	Feature Count
Constitutional descriptors	30
Connectivity descriptors	44
Kappa descriptors	7
Basak descriptors	21
E-state descriptors	245
Burden descriptors	32
Moreau-Broto autocorrelation descriptors	32
Moran autocorrelation descriptors	32
Geary autocorrelation descriptors	32
Molecular property descriptors	6
MOE-type descriptors	60

1.3 Hyperparameter Tuning

The hyperparameters of all methods in paper were optimized on the validation pairs and performance is measured on test pairs. The hyperparameters with their corresponding ranges for baseline methods are given at Table tab:mmparams, 6, 7 and 8.

Table 5. Hyperparameters for MatchMaker. The input data is normalized by *tanh_norm*. *tanh_norm* first standardizes, applies hyperbolic tangent and standardizes again.

Hyperparameter	Tuned values
preprocessing	tanh_norm
number of hidden layer (for each sub-network)	2, 3
number of neurons (for each sub-network)	512, 1024, 2048, 4096
dropout	0.5
input dropout	0.2
learning rate	0.0001

Table 6. Hyperparameters for DeepSynergy. The input data is normalized either by *tanh_norm* or *tanh* as given at *Preuer et al.*. *tanh* first standardizes and then applies hyperbolic tangent. *tanh_norm* first standardizes, applies hyperbolic tangent and standardizes again.

Hyperparameter	Tuned values
preprocessing	tanh_norm, tanh
number of hidden layer	2, 3
number of neurons	512, 1024, 2048, 4096
dropout	0.5
input dropout	0.2
learning rate	0.0001

Table 7. Hyperparameters for TreeCombo. The input data is normalized with *tanh_norm*. The number of estimator parameter is set to 1000 as given at *Janizek et al.* Maximum tree depth and learning rate ranges used same as *Janizek et al.*

Hyperparameter	Tuned values
preprocessing	tanh_norm
maximum tree depth	4, 6, 8, 10, 12
learning rate	0.05, 0.10, 0.15
The number of estimators	1000

Table 8. Hyperparameters for Random Forest. The input data is normalized with *tanh_norm*.

**Hyperparameter**	**Tuned values**
preprocessing	tanh_norm
maximum tree dept	4, 6, 8, 10, 12
the number of estimator	10, 100, 500, 1000
